# Retinal Vascular Diseases Highlighted by Adaptive Optics Ophthalmoscopy

**DOI:** 10.22336/rjo.2025.07

**Published:** 2025

**Authors:** Andrada-Elena Mirescu, Dan George Deleanu, George Baltă, Ioana Teodora Tofolean, Florian Baltă, Irina-Elena Cristescu, Sanda Jurja

**Affiliations:** 1 “Ovidius” University of Constanţa, Constanţa, Romania; 2 “Carol Davila” University of Medicine and Pharmacy, Bucharest, Romania; 3 University Emergency Hospital, Bucharest, Romania; 4 Clinical Emergency Eye Hospital, Bucharest, Romania; 5 Retina Clinic, Bucharest, Romania; 6 County Clinical Emergency Hospital of Constanţa, Constanţa, Romania

**Keywords:** adaptive optics, retinal vascular diseases, adaptive optics, diabetic retinopathy, macular telangiectasia, AO = adaptive optics, SHWS = Shack-Hartmann wavefront sensor, FC = fundus cameras, SLO = scanning laser ophthalmoscopy, OCT = optical coherence tomography, TD = total vessel diameter, LD = lumen diameter, WT = wall thickness, WLR = wall-to-lumen ratio, WCSA = wall cross-sectional area, BCVA = best corrected visual acuity, ROI = regions of interest, DR = diabetic retinopathy, MacTel = macular telangiectasia

## Abstract

**Objective:**

We used adaptive optics ophthalmoscopy to assess retinal microcirculation and photoreceptor parameters in healthy individuals and patients with vascular retinal diseases. This technology enhances optical system resolution to 2 µm by correcting wavefront aberrations, revolutionizing in vivo studies of ocular structures.

**Methods:**

Our study examined the clinical applications of adaptive optics in both healthy individuals and patients with vascular retinal diseases, including nonproliferative diabetic retinopathy, proliferative diabetic retinopathy, and macular telangiectasia (MacTel) type 2.

**Results:**

Our study underlined a higher wall-to-lumen ratio (WLR) value in our patient with proliferative diabetic retinopathy compared to our healthy volunteer. Additionally, we found a positive correlation between WLR and the severity of diabetic retinopathy. Furthermore, cone density was lower in all quadrants with proliferative diabetic retinopathy. For our patient diagnosed with MacTel type 2, the cone mosaic appeared irregular and blurred, with notable cone loss, especially on the temporal side of the macula, consistent with the typical location of MacTel type 2 lesions.

**Discussion:**

Adaptive optics imaging assesses retinal changes in vascular diseases despite acquisition challenges. The obtained images aid in tracking diabetic retinopathy progression and detecting early MacTel Type 2 changes. Our study highlighted vascular and photoreceptor changes, quantifying these parameters to enhance understanding of these vascular diseases.

**Conclusions:**

Adaptive optics imaging is an advanced technique that provides high-resolution visualization of the microstructure of retinal vasculature and photoreceptors. This technology enhances our understanding of healthy and vascular retinal conditions, aiding diagnosis, monitoring, and prognosis.

## Introduction

Until recently, assessing the retina at the cellular level was only achievable through histological techniques [1]. Direct visualization of individual cells in the living retina could transform our understanding of retinal structure and function in healthy and diseased retinas [2]. Retinal imaging technology previously lacked sufficient resolution due to optical aberrations within the eye. The introduction of adaptive optics (AO) technology enhanced optical system resolution to 2 µm by correcting wavefront aberrations, revolutionizing in vivo studies of eye structures [1].

AO was initially implemented in astronomical telescopes to correct optical wavefront aberrations caused by atmospheric turbulence, enhancing the clarity of images of distant celestial objects [1]. Since then, the total optical aberrations of the human eye have been measurable using a Shack-Hartmann wavefront sensor (SHWS), a crucial innovation in the advancement of AO technology [2]. A wavefront corrector counteracts the aberrations detected by the SHWS. The most commonly used type is the deformable mirror, which adjusts the shape of its surface, thus correcting aberrations to obtain high-resolution retinal images [3,4].

AO technology is fundamentally a method for correcting optical aberrations. It does not generate images on its own; therefore, it must be incorporated into existing retinal imaging systems such as fundus cameras (FC), scanning laser ophthalmoscopy (SLO), and optical coherence tomography (OCT). The AO-FC called rtx1 (Imagine Eyes, Orsay, France) is the first commercially available system. This camera features high lateral resolution (1.6 microns) and a rapid image acquisition time of 4 seconds, during which 40 individual images are captured. The short capture duration minimizes disturbances caused by eye movements. Additionally, AO-FC offers a more cost-effective and accessible alternative than AO-OCT and AO-SLO [1]. The limitations of AO-FC include lower axial image resolution and reduced contrast compared to other imaging devices [2, 5].

AO facilitates non-invasive, in vivo retina imaging at a microscopic level, enabling the detailed analysis of individual structures such as photoreceptors, blood vessels, nerve fibers, ganglion cells, and the lamina cribrosa [1]. Cone photoreceptors were among the first retinal cells to be visualized and quantified using AO technology [2]. The primary photoreceptor parameters analyzed through AO imaging include cone density, spacing, reflectivity, and regularity [6,7]. Once the cells are identified, various metrics can be automatically analyzed [6]. Photoreceptor density is determined by dividing the number of cones in a given region by its area. Recent studies demonstrated that age and axial length are essential for assessing cone density [8]. The distances between neighboring cells are utilized for spacing parameters to create a Voronoi map of the cone mosaic [7]. Voronoi analysis can also offer insights into the uniformity of the cone mosaic [9]. Since cones are typically arranged in a hexagonal lattice pattern, cone regularity is defined by the proportion of cones with exactly six neighboring cells [1].

To evaluate vascular morphology, the following parameters are measured: total vessel diameter (TD), lumen diameter (LD), and wall thickness (WT). The wall-to-lumen ratio (WLR) is also calculated as the ratio of vessel WT to LD. At the same time, the wall cross-sectional area (WCSA) represents the relationship between LD and TD [10]. Normal subjects’ lumen and total vessel diameters demonstrated a strong linear correlation across various vessel sizes [11]. As a result, WLR can be highly predictable for a specific vessel size, and deviations from this relationship may serve as a biomarker for disease [11].

## Methods

This study was conducted per the Declaration of Helsinki and approved by the Ethics Committee of Ponderas Academic Hospital, Bucharest, Romania, and by the Ethics Committee of “Ovidius” University of Constanţa, Romania. All patients included in the study signed the written informed consent.

Our study explored adaptive optics’ clinical applications in healthy individuals and patients with vascular retinal diseases (nonproliferative or proliferative diabetic retinopathy and macular telangiectasia type 2). Our data highlighted how this state-of-the-art imaging technique offers highly detailed insights into the microstructure of the retinal vasculature and photoreceptors.

After a comprehensive ophthalmological examination (including best-corrected visual acuity - BCVA, refraction, intraocular pressure assessment, and slit-lamp evaluation of both the anterior and posterior eye segments), high-resolution adaptive optics imaging and axial length measurement were also conducted.

Adaptive optics retinal camera rtx1™ (Imagine Eyes, France) was used for the microcirculation and the photoreceptors assessment. The software allowed for refractive error correction and applied a standardized focus depth of 200 µm for microvascular measurements and 70 µm for photoreceptor measurements in all patients. The retinal microvasculature was analyzed using AOdetect™ Wall (Imagine Eyes, France), while photoreceptor density was quantified with AOdetect™ Mosaic (Imagine Eyes, France). Axial length was also considered for both microvascular and photoreceptor analyses. Several vascular parameters were analyzed, including total vessel diameter (TD), lumen diameter (LD), mean wall thickness (WT), wall-to-lumen ratio (WLR), and the cross-sectional area of the vascular wall (WCSA). Three selected regions of interest (ROI) (each measuring 100 μm in width and height) were examined. Regarding the photoreceptors, images were captured with the fixation point centered in the fovea. Four regions of interest (ROI) were examined across all quadrants, each located 1 degree from the fovea (each measuring 100 μm in width and height). The axial length was measured using the IOL Master 700 (Carl Zeiss Meditec, Germany).

## Results

**Fig. 1:**
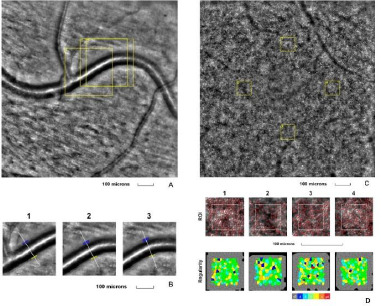
Adaptive optics imaging in a healthy volunteer patient. **A**. Image of the retinal arteriole obtained using an adaptive optics camera, measured in three different regions of interest (highlighted by yellow squares in the image). **B**. The three regions of interest were analyzed separately. **C**. Image of the cone mosaic obtained using an adaptive optics camera, measured in four different regions of interest (highlighted by yellow squares in the image): superior (first), nasal (second), inferior (third), and temporal (fourth). **D**. Red squares correspond to the identified cones, showing the cones in all four regions of interest (top image). Voronoi diagrams of all four regions of interest illustrate the cone regularity index (bottom image).

The first examined eye was the left eye of a 29-year-old healthy female patient with no known pathological conditions and no ongoing treatments. Her left eye BCVA was 1.0 (decimal scale) at the time of examination, showing no anterior or posterior pole modifications. The retinal arteriole and cone mosaic images appeared normal (**Fig. 1**).

**Fig. 2:**
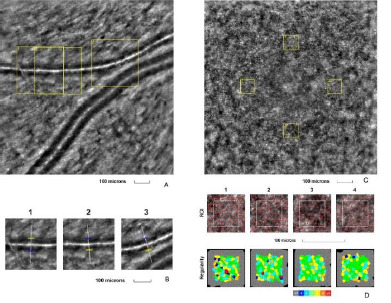
Adaptive optics imaging in a nonproliferative diabetic patient. **A**. Image of the retinal arteriole obtained using an adaptive optics camera, measured in three different regions of interest (highlighted by yellow squares in the image). **B**. The three regions of interest were analyzed separately. **C**. Image of the cone mosaic obtained using an adaptive optics camera, measured in four different regions of interest (highlighted by yellow squares in the image): superior (first), temporal (second), inferior (third), and nasal (fourth). **D**. Red squares correspond to the identified cones, showing the cones in all four regions of interest (top image). Voronoi diagrams of all four regions of interest illustrate the cone regularity index (bottom image).

The second eye examined was the right eye of a 44-year-old female, with a BCVA of 1.0 (decimal scale) at the time of examination, diagnosed with nonproliferative diabetic retinopathy. The patient had been suffering from type 2 diabetes mellitus for the past 15 years. Both the retinal arteriole and cone mosaic images exhibited changes (**Fig. 2**).

**Fig. 3:**
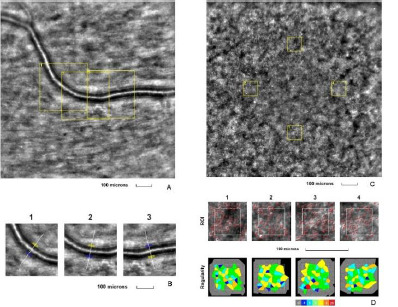
Adaptive optics imaging in a proliferative diabetic patient. **A**. Image of the retinal arteriole obtained using an adaptive optics camera, measured in three different regions of interest (highlighted by yellow squares in the image). **B**. The three regions of interest were analyzed separately. **C**. Image of the cone mosaic obtained using an adaptive optics camera, measured in four different regions of interest (highlighted by yellow squares in the image): superior (first), temporal (second), inferior (third), and nasal (fourth). **D**. Red squares correspond to the identified cones, showing the cones in all four regions of interest (top image). Voronoi diagrams of all four regions of interest illustrate the cone regularity index (bottom image).

The third eye examined was the right eye of a 44-year-old female, with a BCVA of 1.0 (decimal scale) at the time of examination. She had a prior diagnosis of proliferative diabetic retinopathy but had not received laser or anti-vascular endothelial growth factor treatment in the past year. The patient had been living with type 1 diabetes mellitus for the past 28 years. Modifications were observed in the retinal arteriole and cone mosaic images (**Fig. 3**).

**Fig. 4:**
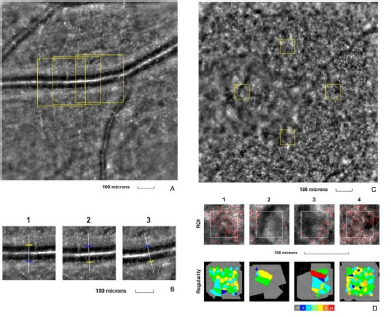
Adaptive optics imaging in a patient with macular telangiectasia type 2. **A**. Image of the retinal arteriole obtained using an adaptive optics camera, measured in three different regions of interest (highlighted by yellow squares in the image). **B**. The three regions of interest were analyzed separately. **C**. Image of the cone mosaic obtained using an adaptive optics camera, measured in four different regions of interest (highlighted by yellow squares in the image): superior (first), temporal (second), inferior (third), and nasal (fourth). **D**. Red squares correspond to the identified cones, showing the cones in all four regions of interest (top image). Voronoi diagrams of all four regions of interest illustrate the cone regularity index (bottom image).

The fourth examined eye was the right eye of a 67-year-old female, with a BCVA of 0.9 (decimal scale) at the time of examination. At the time of assessment, she was diagnosed with macular telangiectasia type 2. The retinal arteriole and cone mosaic parameters were measured (**Fig. 4**).

**Table 1** presents the values of the arteriole parameters for each patient, with each parameter representing the average of three consecutive measurements. The WLR value of our patient with proliferative diabetic retinopathy was higher compared to that of our healthy volunteer. Additionally, we observed a positive correlation between WLR and the severity of DR, with the value being higher in our patient with proliferative diabetic retinopathy than in the one with nonproliferative diabetic retinopathy (**Table 1**).

**Table 1:** Adaptive optics vascular parameters

Adaptive optics vascular parameters	Healthy volunteer	Nonproliferative diabetic retinopathy patient	Proliferative diabetic retinopathy patient	Macular telangiectasia type 2 patient
Lumen diameter (VD) (μm)	87,4	59,7	53,26	79,76
Total vessel diameter (TD) (μm)	107,7	79,33	75,33	98,83
Wall thickness (WT) (μm)	10,18	9,88	11,05	10,05
Wall to lumen ratio (WLR)	0,23	0,33	0,41	0,25
Wall cross-sectional area (WCSA) (μm^2^)	3114,24	2160,93	2232,54	2834,66

The cone mosaic values for each patient are presented in **Table 2**, with each parameter measured in four regions of interest, each situated 1 degree from the fovea across all quadrants. A lower cone density was observed in all quadrants of our patient with proliferative diabetic retinopathy compared to our healthy volunteer. Additionally, we noted a progressive decline in cone density as DR severity increased. For our patient diagnosed with MacTel type 2, the cone mosaic appeared irregular and blurred, with significant cone loss, particularly on the temporal side of the macula, aligning with the location of MacTel type 2 lesions (**Table 2**).

**Table 2:** Adaptive optics cone mosaic parameters

Adaptive optics cone mosaic parameters	Healthy volunteer	Nonproliferative diabetic retinopathy patient	Proliferative diabetic retinopathy patient	Macular telangiectasia type 2 patient
**Superior quadrant**				
-density (mm^2^)	29 333	23 618	13 961	18 072
-spacing (μm)	6,46	7,26	9,27	8,04
-regularity (%)	90,09	79,75	84,78	93,55
**Temporal quadrant**				
-density (mm^2^)	27 623	23 104	10 930	4 198
-spacing (μm)	6,71	7,26	10,28	14,8
-regularity (%)	91,26	90,59	84,21	75
**Inferior quadrant**				
-density (mm^2^)	27 275	20 732	13 512	8 026
-spacing (μm)	6,67	7,79	9,31	10,74
-regularity (%)	84,62	95,89	88,24	87,5
**Nasal quadrant**				
-density (mm^2^)	19 356	21 821	11 677	19 952
-spacing (μm)	7,98	7,57	9,93	7,77
-regularity (%)	86,67	89,02	88,37	89,33

## Discussion

One of the primary applications of AO imaging is the evaluation of photoreceptors and blood vessels in diabetic patients. However, AO retinal imaging in patients with diabetes mellitus can be challenging due to diabetic complications such as impaired mydriasis, cataract, and vitreous hemorrhage [12]. Zaleska-Zmijewska et al. evaluated cone parameters and retinal vessels using AO-FC in diabetic patients. Their study revealed a significantly lower cone density in diabetic retinopathy (DR) patients compared to the control group, with a progressive decline in cone density and regularity as DR severity increased. Additionally, arterial walls were notably thicker in the DR group than in the control group [12]. Ueno et al. evaluated the morphological parameters of retinal vessels using AO-FC in patients with type 2 diabetes mellitus. The WLR was significantly greater in the proliferative diabetic retinopathy group compared to all other groups. The WLR exhibited a positive correlation with the severity of DR, duration of disease, systolic blood pressure, and systemic hypertension [13].

In our patient with proliferative diabetic retinopathy, cone density was lower across all quadrants than in our healthy volunteer. Furthermore, we observed a progressive decline in cone density with increasing DR severity. In the cases above, we also noted that our patient with proliferative diabetic retinopathy had a higher WLR value than the healthy volunteer. Additionally, our data showed a positive correlation between WLR and the severity of diabetic retinopathy and the disease duration.

Idiopathic macular telangiectasia (MacTel) is traditionally regarded as a vascular disorder affecting the juxtafoveal retinal capillaries. Emerging evidence indicates that neuronal alterations may arise in the early stages of the disease [14]. Song et al. aimed to investigate early photoreceptor changes in MacTel type 2 using AO-SLO. Although the less-affected eyes appeared normal or nearly normal clinically, all patients demonstrated a significantly reduced cone density at the foveal center [14]. This shows that loss of cone photoreceptors may occur before detectable vascular changes in MacTel [14]. Jacob et al. demonstrated that the most significant cone loss happened on the temporal side of the macula, aligning with the characteristic location of MacTel 2 lesions [15]. AO imaging revealed an irregular, patchy disturbance of the cone mosaic in MacTel 2 eyes [15]. The study demonstrated that AO imaging can detect early structural changes in the disease process before significant vision loss, making it a potential tool for early diagnosis and disease monitoring [15].

In our MacTel type 2 patient, the cone mosaic appeared irregular and blurred, with significant cone loss, particularly on the temporal side of the macula, corresponding to the location of MacTel type 2 lesions.

Studies with a larger patient population could help achieve a better understanding of both vascular parameters and cone.

## Conclusion

In conclusion, adaptive optics imaging is a cutting-edge technique that provides highly detailed visualization of the microstructure of the retinal vasculature and photoreceptors. This advanced imaging method improves our understanding of normal and pathological retinal vascular conditions. The comprehensive data gathered can enhance our understanding of disease pathology, support more precise diagnoses, and provide valuable insights for monitoring and prognosing retinal disorders.
